# Effectiveness of Stapedotomy in Improving Audibility and Quality of Life in Patients with Unilateral Otosclerosis – A Retrospective Study

**DOI:** 10.1055/s-0045-1811953

**Published:** 2026-03-17

**Authors:** Andrzej Pastuszak, Elżbieta Gos, Aleksandra Kolodziejak, Łukasz Plichta, Piotr Henryk Skarzynski

**Affiliations:** 1 Otorhinolaryngosurgery Clinic, World Hearing Center, Institute of Physiology and Pathology of Hearing, Warsaw, Poland; 2 Department of Teleaudiology and Screening, World Hearing Center, Institute of Physiology and Pathology of Hearing, Warsaw, Poland; 3 Institute of Sensory Organs, Kajetany, Warsaw, Poland

**Keywords:** stapedotomy, otosclerosis, quality of life

## Abstract

**Objective:**

To assess the quality of life in patients with unilateral otosclerosis treated surgically with stapedotomy. Both objective indicators (hearing thresholds and air-bone gap) and subjective patient assessment of quality of life were considered.

**Methods:**

We included 84 patients with unilateral otosclerosis who underwent stapes surgery. There were 24 men and 60 women, aged between 22 and 73 (mean = 44.1 ± 10.5) years. Pure tone audiometry (frequency range: 0.125–8 kHz) was performed preoperatively and 6 and 12 months postoperatively. Quality of life was assessed with the Assessment of Quality of Life – 8 Dimensions (AQoL-8D) questionnaire.

**Results:**

The preoperative air-conduction hearing thresholds were on average 56.6 dB HL and improved significantly to 31.3 dB HL 6 months after stapes surgery. Air-bone gap before surgery was on average 29.8 dB HL and improved significantly to 9.9 dB 6 months after stapes surgery. Hearing thresholds were stable for 12 months after stapes surgery. Overall quality of life significantly increased after surgery, with improvements occurring in several domains: *independent living* , *senses* , *mental health* , *coping* , and *relationships* .

**Conclusion:**

Stapedotomy is an effective treatment for patients with unilateral otosclerosis. As well as improving hearing thresholds, it significantly enhances overall quality of life.

## Introduction

Approximately 430 million people, over 5% of the global population, suffer from various types of hearing problems. Most of these individuals require specialized care, treatment, and rehabilitation. Depending on the type and degree of hearing loss, patients may undergo surgical treatment or use hearing aids. 1

Hearing disorders significantly reduce the quality of life of those affected by it. Hearing loss negatively impacts the ability to understand speech, leading to communication difficulties. It also worsens the patient's overall functioning on physical, emotional, and social levels. 2 3 Many factors can contribute to hearing issues, one of which is otosclerosis, a middle ear condition diagnosed in approximately 10% of patients with a hearing disorder. 4 Otosclerosis is characterized by pathological processes occurring in the temporal bone, leading to abnormal remodeling of the labyrinth. 5 The process involves resorption of bone tissue, followed by recalcification and vascular proliferation within the temporal bone. 6 7 The result is stiffening of the stapes footplate and, consequently, the entire ossicular chain, causing conductive hearing loss. Otosclerosis is a progressive condition, and bone remodeling can extend beyond the stapes footplate to the cochlea, 8 leading to increased bone-conduction (BC) thresholds and introducing a sensorineural component to the hearing loss. Otosclerosis typically affects both ears, with only 20% of cases being unilateral. 9 10

Otosclerosis can be diagnosed intraoperatively. Audiometric tests and imaging (particularly high-resolution computed tomography [HRCT] scans) can help guide the diagnosis. 7 The etiopathogenesis of otosclerosis is not entirely understood, and it is currently considered multifactorial. Research suggests that genetic and environmental factors play a significant role in the development of the disease. The literature indicates that viral infections, especially measles, may contribute to their onset. 6 11 12

In addition to hearing loss, patients with otosclerosis may experience tinnitus and occasional dizziness. These symptoms can negatively affect the patient's physical and mental health, as well as social functioning. 13

Since there is no other treatment for otosclerosis, stapes surgery is the choice of treatment. Previously, stapedectomy was used, but the current method is stapedotomy; both aim to improve hearing and slow disease progression. The current procedure is considered effective if it reduces the cochlear reserve to within 10 dB (air-bone gap, ABG) and improves air-conduction (AC) thresholds. 14

Although reporting a surgical outcome provides information about hearing improvement, it does not indicate the impact of the surgery on the patient's quality of life. From the patient's perspective, a successful outcome means improved quality of life and better functioning in daily activities. 15 It is, therefore, helpful to evaluate the surgical procedure used in treating otosclerosis not just through objective tests but also through the patient's own assessment.

In recent years, health-related quality of life (HRQoL) has become increasingly important in healthcare. This concept aligns with a biopsychosocial approach in which health is understood not only as the absence of disease but as complete physical, mental, and social wellbeing. 16 17 Therefore, the subjective benefits that patients experience in daily life are important, and when assessing the effectiveness of surgery for otosclerosis, both audiometric results and the patient's quality of life should be considered.

In this context, some authors have suggested that audiometric assessments do not always match the patient's subjective assessment of quality-of-life changes. 18 19 20 The difference may arise because hearing loss tends to correlate more closely with mental health and quality of life than with the degree of hearing impairment measured audiometrically. 20

To assess quality of life, standardized questionnaires are typically used. They give a multi-faceted measure of a patient's functioning and the impact of disease and treatment on their overall wellbeing. A common tool for assessing health-related quality of life in otolaryngology patients is the Assessment of Quality of Life – 8 Dimensions (AQoL-8D) questionnaire. Many otolaryngological conditions, such as hearing loss, dizziness, or tinnitus, often lead to long-term disability, which is known to affect mental health and social functioning. The AQoL-8D questionnaire is a suitable tool for examining health-related quality of life in otolaryngology patients, 13 21 and it was used in this study.

## Objective

The objective of the present study was to assess audibility and quality of life in patients with unilateral otosclerosis treated surgically through stapedotomy. The analysis included both objective indicators (hearing thresholds and cochlear reserve levels) and subjective patient evaluations using the AQoL-8D questionnaire.

## Methods

### Inclusion Criteria

The study included 84 patients with unilateral otosclerosis scheduled for stapedotomy surgery at the Institute of Physiology and Pathology of Hearing, Warsaw. The following inclusion criteria were applied:

- age over 18 years;
- intraoperatively confirmed stapes fixation of one ear;
- results from pre- and postoperative pure tone audiometry;
- AQoL-8D questionnaire assessments before and after surgery.

The study received approval from the institutional Bioethics Committee (IFPS/6/2022) and was conducted in accordance with the Declaration of Helsinki. All patients provided informed consent to participate in the study.

### Audiometric Assessment

Patients underwent pure tone audiometry (PTA) to evaluate AC thresholds (frequency range: 0.125–8 kHz) and BC thresholds (0.25–4 kHz). Testing was performed by an experienced technician in an audiometric booth, using the Madsen Itera II audiometer (GN Otometrics) with calibrated H-39P headphones (Telephonics).

### AQoL-8D

The AQoL-8D is a questionnaire used to assess HRQoL in patients undergoing otolaryngological treatment. It is based on the World Health Organization (WHO) definition of health as a state of complete physical, mental, and social wellbeing, not merely the absence of disease or disability. 22

The AQoL-8D was developed by a research team at the Centre for Health Economics at Monash University, in Australia. 13 23 A Polish adaptation of the AQoL-8D questionnaire has been carried out by Obrycka et al. ^21^ The tool was self-administered by the patients in paper format. The responses were scored using the official scoring algorithm, which is publicly available on the AQoL website ( https://www.aqol.com.au ).

The AQoL-8D consists of 35 items covering 8 dimensions: *independence* , *pain* , *senses* , *mental* *health* , *happiness* , *coping* , *relationships* , and *self-worth* . Three of these ( *independence* , *pain* , and *senses* ) relate to the physical dimension of quality of life, while the remaining five address the psychosocial dimension. During the AQoL-8D validation, Obrycka et al. ^21^ demonstrated that all items were highly relevant for measuring HRQoL in patients undergoing otolaryngological treatment. Furthermore, the authors showed a strong correlation between the AQoL-8D utility index (UI) and the Short-Form 6 Dimensions (SF-6D), confirming that both questionnaires can be used to assess HRQoL.

Postoperative hearing test results are presented, along with measures of the impact on the patients' daily lives, an approach that aligns with modern trends in patient-centered medicine, which emphasize individual expectations and needs. The analysis is based on patient data from audiometric (PTA) and questionnaire (AQoL-8D) assessments conducted preoperatively and at 6 and 12 months postoperatively.

### Surgery

All surgeries for patients diagnosed with unilateral otosclerosis were performed by an experienced otosurgeon, Prof. Henryk Skarżyński, at the Otolaryngology Clinic of the Institute of Physiology and Pathology of Hearing, Kajetany, Poland. The procedure was conducted under general anesthesia, using a transcanal approach to access the middle ear. An incision was made on the posterior wall of the external auditory canal, and the skin and tympanomeatal flap were dissected to identify the chorda tympani. If necessary, the posterior-superior wall of the external auditory canal was widened to improve access to the oval window niche. The mobility of the ossicular chain was then assessed. In most cases, the malleus and incus showed normal mobility (but restricted in the malleus for one patient and in the incus for six patients), whereas the stapes were immobile. The incudostapedial joint was disconnected, and the stapes tendon delicately cut using micro-scissors. After removing the stapes superstructure, a hole was drilled in the stapes footplate using a low-speed microdrill with a 0.6-mm diamond burr. The prosthesis was inserted into the footplate hole, attached to the long process of the incus, and sealed with a blood clot. After repositioning and securing the tympanomeatal flap (using tissue glue), a dressing was placed in the external auditory canal consisting of a foil, cotton wool, and two wicks. To rule out other middle or inner ear pathologies, patients underwent HRCT of the temporal bones prior to surgery.

### Participants

There were 84 patients (24 men and 60 women) with unilateral otosclerosis who underwent stapes surgery. Their ages ranged from 22 to 73 (mean = 44.1 ± 10.5) years.

### Statistical Analysis

Statistical description included the percentage distributions for categorical variables and descriptive statistics for quantitative variables. The assumption of normality for hearing thresholds and quality of life was checked using a Kolmogorov-Smirnov test. A Wilcoxon test was applied to compare hearing outcomes and quality of life before and after surgery. The relationship between hearing thresholds and quality of life was assessed using Spearman's rank correlation coefficients. Statistical significance was taken as a *p* -value < 0.05. Data analysis was conducted using IBM SPSS Statistics for Windows (IBM Corp.) version 24.0.

## Results

### Clinical Characteristics of the Patients

There were 77 patients who underwent stapedotomy, 3 restapedotomy, and 4 revision surgery. Most of the patients (73%) had tinnitus, while vertigo was less frequent (24%). The duration of hearing loss averaged 9.3 years. Detailed clinical characteristics of the patients are set out in Table 1 .

**Table 1:** Clinical characteristics of the patients

		*N* = 84
Duration of hearing loss (years)	Range	1–39
	mean	9.3(± 7.5)
Operated ear: n (%)	Right	46 (55)
	Left	38 (45)
Surgery: n (%)	Stapedotomy	77 (92)
	Restapedotomy	3 (4)
	Revision	4 (5)
Tinnitus: n (%)	Yes	61 (73)
	No	23 (27)
Vertigo: n (%)	Yes	20 (24)
	No	64 (76)
Other surgeries in the operated ear: n (%)	Yes	10 (12)
	No	74 (88)
Malleus mobility: n (%)	Normal	83 (99)
	Limited	1 (1)
Incus mobility: n (%)	Normal	78 (93)
	Limited	6 (7)
Stapes mobility: n (%)	Normal	0 (0)
	Limited	84 (100)

### Hearing Thresholds and Air-Bone Gap Before and 6 Months After Stapes Surgery

Preoperatively, the AC hearing thresholds ranged from 26.2 to 107.5 (mean = 56.6 ± 16.7) dB HL. After stapes surgery, they were much improved, (range = 13.8–81.7 dB HL; mean = 31.3 ± 12.7 dB HL). The change was statistically significant ( *Z* = 7.89; *p* < 0.001).

Preoperatively, the BC hearing thresholds were 6.3 to 62.5 (mean = 26.8 ± 12.4) dB HL. After surgery, they were in the range 5 to 67.5 (mean = 21.5 ± 11.2) dB HL. The change was statistically significant ( *Z* = 5.69; *p* < 0.001).

**Fig. 1:**
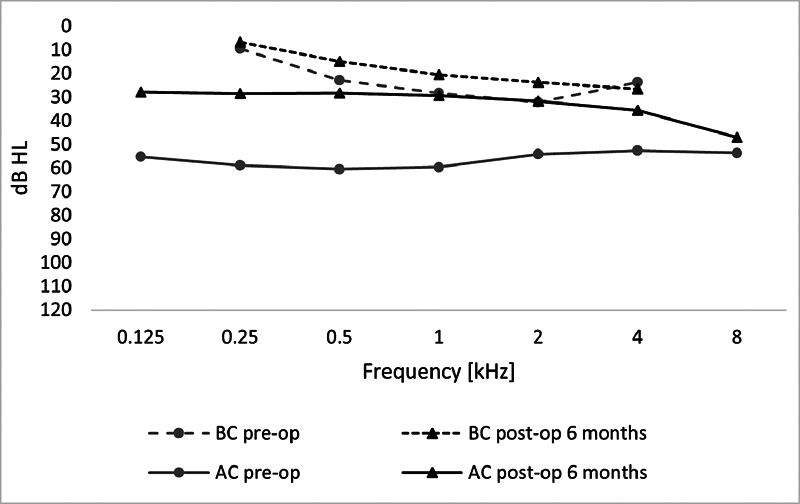
Hearing thresholds before and 6 months after stapes surgery.

The ABG before surgery was of 8.8 to 57.5 (mean = 29.8 ± 9.7) dB HL. After surgery, the ABG was in the range of 1.3 to 51.7 (mean = 9.9 ± 5.4) dB HL. The change was statistically significant ( *Z* = 7.92; *p* < 0.001). Hearing thresholds from 0.125 to 8 kHz are shown in Fig. 1 .

**Fig. 2:**
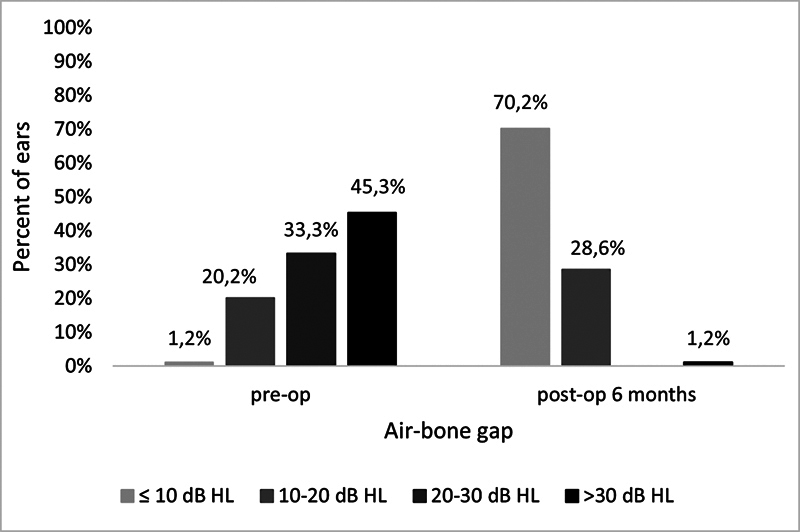
Air-bone gap before and 6 months after stapes surgery.

The ABG before surgery was lower than 10 dB in 1 patient, from 10 to 20 dB in 17 patients, from 20 to 30 dB in 28 patients, from 30 to 40 dB in 29 patients, from 50 to 60 dB in 7 patients, and from 60 to 70 dB in 2 patients. Six months after surgery, the ABG was lower than 10 dB in 59 patients, from 10 to 20 dB in 24 patients, and from 60 to 70 dB in 1 patient. The percentage distribution is shown in Fig. 2 .

### Quality of Life Before and 6 Months After Stapes Surgery

Table 2 shows quality of life before and 6 months after stapes surgery.

**Table 2:** Descriptive statistics for quality of life measured with the AQoL-8D before and 6 months after stapes surgery

	Before stapes surgery				6 months after stapes surgery			
	*Min.*	*Max.*	*Mean*	*SD*	*Min.*	*Max.*	*Mean*	*SD*
Independent living	0.76	1	0.93	± 0.07	0.62	1	0.94	± 0.07
Pain	0.47	1	0.86	± 0.16	0.33	1	0.84	± 0.17
Senses	0.37	0.97	0.74	± 0.14	0.35	0.97	0.79	± 0.14
Mental health	0.32	0.96	0.61	± 0.12	0.32	1	0.63	± 0.13
Happiness	0.56	0.93	0.77	± 0.11	0.50	1	0.78	± 0.12
Coping	0.48	1	0.80	± 0.11	0.51	1	0.83	± 0.11
Relationships	0.49	1	0.77	± 0.14	0.49	1	0.81	± 0.14
Self-worth	0.46	1	0.82	± 0.14	0.52	1	0.84	± 0.13
Physical superdimension	0.39	0.98	0.71	± 0.15	0.25	0.95	0.74	± 0.15
Psychosocial superdimension	0.12	0.90	0.41	± 0.16	0.10	0.89	0.45	± 0.18
Overall quality of life	0.34	0.98	0.72	± 0.16	0.30	0.99	0.76	± 0.16

**Abbreviations:** AQoL-8D, Assessment of Quality of Life –8 Dimensions; Max., maximum score; Min., minimum score; SD, standard deviation.

The overall quality of life significantly improved after stapes surgery ( *Z* = 3.07; *p* = 0.002). Significantly higher quality of life was also found in the dimensions of *independent living* ( *Z* = 2.18; *p* = 0.029), *senses* ( *Z* = 2.18; *p* = 0.029), *mental health* ( *Z* = 2.18; *p* = 0.029), *coping* ( *Z* = 2.18; *p* = 0.029), and *relationships* ( *Z* = 2.18; *p* = 0.029), as well as in the *physical superdimension* ( *Z* = 2.18; *p* = 0.029) and the *psychosocial superdimension* ( *Z* = 2.18; *p* = 0.029).

### Relationship Between Hearing Thresholds and Quality of Life

A statistically significant correlation between hearing thresholds and quality of life was found before stapes surgery as well as 6 months after stapes surgery ( Table 3 ).

**Table 3:** Correlations involing hearing thresholds and quality of life before and 6 months after stapes surgery

	Before stapes surgery			6 months after stapes surgery		
Variables	AC	BC	ABG	AC	BC	ABG
Independent living	–0.37**	–0.46***	–0.01	–0.21	–0.18	–0.06
Pain	–0.17	–0.23*	0.03	–0.26*	–0.28*	0.07
Senses	–0.30**	–0.34**	–0.05	–0.48***	–0.42***	–0.21
Mental health	–0.18	–0.24*	0.02	–0.09	–0.12	0.06
Happiness	–0.18	–0.22*	0.01	–0.27*	–0.29*	0.02
Coping	–0.07	–0.11	0.11	–0.14	–0.11	–0.09
Relationships	–0.12	–0.12	0.07	–0.04	–0.04	0.03
Self-worth	0.03	–0.16	0.22	–0.11	–0.11	0.01
Physical superdimension	–0.34**	–0.40***	–0.03	–0.47***	–0.44***	–0.08
Psychosocial superdimension	–0.14	–0.20	0.09	–0.14	–0.15	0.02
Overall quality of life	–0.20	–0.26*	0.06	–0.25*	–0.25*	0.01

**Abbreviations** : AC, air conduction; ABG, air-bone gap; BC, bone conduction.

**Notes** : * *p* < 0.05; ** *p* < 0.01; *** *p* < 0.001.

Before surgery, hearing thresholds were correlated with quality of life in the dimensions *independent living* , *senses* , and *physical superdimension* (for AC) and with *independent living* , *pain* , *senses* , *mental health* , *happiness* , *physical superdimension* , and *overall quality of life* (for BC). The ABG was not correlated with quality of life.

Six months after stapes surgery, hearing thresholds were correlated with quality of life in the dimensions of pain, *senses* , *happiness* , *physical superdimension* , and *overall quality of life* (for AC) and with *pain* , *senses* , *happiness* , *physical superdimension* , and *overall quality of life* (for BC). Again, the ABG was not correlated with quality of life. Correlations between hearing thresholds and quality of life were negative, meaning that the lower (better) the thresholds, the higher the quality of life. Correlations were stronger in the 6-month follow-up, when the patients' hearing was better than before surgery.

## Hearing Outcomes one Year After Stapes Surgery

One year after stapes surgery, hearing outcomes were available for 50 patients. The AC thresholds ranged from 15 to 80 (mean = 31.8 ± 12.8) dB HL. The BC thresholds ranged from 7.5 to 50 (mean = 21.7 ± 10.5) dB HL. The ABG ranged from 1 to 42.5 (mean = 10.1 ± 5.8) dB HL. The ABG was lower than 10 dB in 33 patients (66%), from 10 to 20 dB in 16 patients (32%), and from 50 to 60 dB in 1 patient (the latter being the same patient whose ABG was large at the 6-month follow-up). In other words, hearing outcomes were stable between 6 and 12 months after stapes surgery.

## Quality of Life one Year after Stapes Surgery

One year after the stapes surgery, the outcomes of the AQoL-8D were available for 52 patients. The mean overall quality of life was of 0.79 ± 0.15. For *physical superdimension* , the mean results were of 0.77 ± 0.14, including *independent living* (mean = 0.96 ± 0.07), *pain* (mean = 0.86 ± 0.16), and *senses* (mean = 0.81 ± 0.10). For *psychosocial superdimension* , the mean results were of 0.48 ± 0.18), including *mental health* (mean = 0.64 ± 0.12), *happiness* (mean = 0.79 ± 0.12), *coping* (mean = 0.85 ± 0.12), *relationships* (mean = 0.83 ± 0.11), and *self-worth* (mean = 0.87 ± 0.11).

## Discussion

The goal of the present study was to evaluate hearing outcomes and quality of life in patients with unilateral otosclerosis who underwent stapedotomy. Before surgery, patients underwent pure-tone audiometry testing. The average hearing threshold for AC in our group was 56.6 dB, while for BC it was 26.8 dB HL. The preoperative ABG averaged 29.8 dB HL.

The literature commonly assesses stapedotomy outcomes by reduction in AC thresholds and by the closure of cochlear reserve to within 10 dB, considering pre- and postoperative BC thresholds as well. 14 24

In our study group, a significant improvement in hearing thresholds was observed to be 6 months postsurgery, reflected in an average AC improvement of 25.3 dB, BC improvement of 5.3 dB, and an overall ABG improvement of 19.9 dB. Six months postsurgery, the cochlear reserve averaged 9.9 dB HL, and 1-year postsurgery it was nearly the same at 10.1 dB HL. These findings indicate a reduction in AC thresholds postsurgery, with a sustained decrease in hearing reserve one year later. Additionally, postoperative improvements in BC thresholds further highlight the positive impact of stapedotomy on patient hearing, resulting in an average ABG level of 10.1 dB HL.

Our results are aligned with findings from other researchers. Studies by Dziendziel et al., 22 Weiss et al., 25 Lailach et al., 18 and Subramaniam et al. 26 report statistically significant reductions in cochlear reserve following stapedotomy. Moreover, Dziendziel et al. 22 and Lailach et al. 18 noted a significant change in BC thresholds. In our study, we also observed a reduction in cochlear reserve. Preoperative ABG averaged 29.8 dB HL, while postoperative ABG averaged 9.9 dB HL, and a statistically significant change was observed. Furthermore, like Dziendziel et al. 22 and Lailach et al., 18 our group experienced changes in BC thresholds. The preoperative BC thresholds averaged 26.8 dB HL, and postoperative levels averaged 21.5 dB HL, indicating a statistically significant change.

Increasingly, HRQoL questionnaires have been used to assess the success of otologic procedures. However, publications evaluating HRQoL remain limited. Health-related quality of life should be an integral part of the therapeutic process, since some authors note that audiometric results do not necessarily correlate with patient-reported quality of life. 18 19 20 27 Furthermore, identical levels of hearing impairment can affect individuals' quality of life differently, and even mild hearing loss may significantly impact some patients. 28 This may be explained by other factors, such as general health, lifestyle, or personality, that influence self-perceived impairment beyond audiological criteria.

Our study found a significant improvement in overall quality of life poststapedotomy (significant improvements were also observed in other domains: *independent living* , *senses* , *mental health* , and *relationships* , as well as the *physical* and *psychosocial superdimensions)* . Thus, stapedotomy proved to be effective in improving both hearing and overall quality of life, covering physical and psychosocial aspects.

No significant differences were observed in the *pain* and *happiness* dimensions, indicating that stapedotomy had a limited impact on pain levels and patients' reported happiness. Weiss et al. (2020) ^25^ found a statistically significant relationship between postoperative cochlear reserve (ABG) and quality of life questionnaire results (Stapesplasty Outcome Test 25 [SPOT-25] and Glasgow Benefit Inventory [GBI]), but not with AC thresholds. Most patients reported subjective benefits from the procedure, as shown by the median GBI score of 25. However, subscales for tinnitus and mental state in the SPOT-25, as well as social support in the GBI, did not correlate with postoperative ABG, suggesting that hearing ability alone does not fully define HRQoL for otosclerosis patients. Psychological factors, such as anxiety or social isolation, may play a significant role, supporting the value of using HRQoL questionnaires to assess stapedotomy outcomes.

Unlike Weiss et al., 25 Lailach et al. 18 found no significant correlation between ABG change and GBI questionnaire results, with GBI scores correlating more closely with postoperative hearing outcomes. Similarly, in our study, cochlear reserve did not correlate with quality of life before or after surgery. Hildebrandt et al. 29 also used the SPOT-25 to evaluate quality of life, noting improvements across all subscales except tinnitus. They found significant correlations between AC thresholds and overall SPOT-25 scores but no significant relationships with subscales on social restrictions, mental state, or tinnitus.

Our findings highlight the value of quality-of-life questionnaires in evaluating the benefits of stapedotomy for the treatment of otosclerosis. For example, Browning et al. (1997) reported that patients with bilateral hearing loss benefit twice as much from stapedotomy as patients with unilateral otosclerosis and normal hearing in the other ear. This underscores the greater challenge of achieving satisfactory outcomes in cases of unilateral hearing loss compared to bilateral hearing loss.

While our study provides valuable insights into the outcomes of stapedotomy for unilateral otosclerosis, there are several limitations to consider. First, a larger group and more diverse group of patients could provide more robust results and help confirm the observed trends. Second, our follow-up period was limited to one year, and longer-term data would be necessary to assess the sustainability of hearing improvement and quality of life changes over time. Additionally, we focused on unilateral otosclerosis patients, and future studies could explore the outcomes in patients with bilateral otosclerosis to compare the effects of stapedotomy in different populations.

## Conclusion

Our results indicate that hearing improvement following surgical intervention had a positive impact on patients' quality of life. The use of the HRQoL questionnaire is beneficial, as it is a valuable tool for assessing the subjective benefits of stapedotomy in treating otosclerosis. We recommend that HRQoL measures should complement audiological assessments to present the results of stapedotomy when assessing the efficacy of this surgical procedure.
